# Retraction Note to: Upregulating MicroRNA-410 or Downregulating Wnt-11 Increases Osteoblasts and Reduces Osteoclasts to Alleviate Osteonecrosis of the Femoral Head

**DOI:** 10.1186/s11671-022-03681-9

**Published:** 2022-04-11

**Authors:** Yukun Yin, Lixiang Ding, Yu Hou, Haoran Jiang, Ji Zhang, Zhong Dai, Genai Zhang

**Affiliations:** 1 Department of Traditional Chinese Medicine, National Cancer Center/National Clinical Research Center for Cancer/Cancer Hospital, Chinese Academy of Medical Sciences and Peking Union Medical College, Beijing, 100021 China; 2 Department of Spine Beijing Shijitan Hospital, Capital Medical University, No.10 Tieyi Road, Yangfangdian, Haidian District, Beijing, 100038 People’s Republic of China; 3 Department of General Medicine, Huanxing Cancer Hospital, Chaoyang District, Beijing, 100005 People’s Republic of China

## Retraction Note: Nanoscale Research Letters 14, 383 (2019) 10.1186/s11671-019-3221-6

The Editors-in-Chief have retracted this article. Concerns have been raised regarding a number of figures, specifically:

- Figure 5B: overlapping features can be seen within and between the “ONFH”, “agomir-NC”, “agomir-miR-410” and “Wnt-11-siRNA” panels.
- Figure 5G: blots can be seen to have highly uniform contrast against a uniform grey background.
- Figure 6C: overlapping features can be seen within and between the “agomir-NC”, “siRNA-NC”, “Wnt-11-siRNA”, “agomir-miR-410+OE-NC” and “agomir-miR-410+OE-Wnt-11” panels.
- Figure 7D: blots can be seen to have uniform contrast against a uniform grey background.
- Figure 8E: blots can be seen to have uniform contrast against a uniform grey background.
- Figure 8F: overlapping features can be seen within and between the “Normal”, “ONFH”, “agomir-NC”, “agomir-miR-410”, “siRNA-NC”, and “agomir-miR-410+OE-NC” panels.

The Editors-in-Chief therefore no longer have confidence in the reliability of the data reported in the article. The authors have not responded to any correspondence from the editor/publisher about this retraction.

